# 

**Published:** 1883-11-20

**Authors:** 


### SOUTHERN MEDICAL RECORD:

A MONTHLY JOURNAL OF PRACTICAL MEDICINE.

Terms: $2.00 per Annum, in Advance; 4 Copies $6.00; Single Copies 20 Cents.

*"QUICQUID PRÆCIPIES ESTO BREVIS."*

### TABLE OF CONTENTS.

ORIGINAL AND SELECTED ARTICLES.

Diphtheria, by Charles J. Borroughs, M.D., of Georgia, 401. Infant Feeding, trnaslated from the French by Dr E. Van Goidtsnoven, for the Southern Medical Record, 406. Tonsillitis and its Treatment, by L B. Dawley, M.D., of New York, 407. Quinium Salts, by R. Rother, M.D., 410. The Hot Water Treatment, 412. On the Dry Dressing Treatment of Wounds, by Samson Gamgee, M.D., 415. The Use of Water in the Dietary of Young Children, by Charles Remsen, M D., 417. Gonorrhœa Easily Cured, by Z T. Dellenbaugh, M.D., of Cleveland, Ohio, 419. The Radical Cure of Varicocele, etc., by H. C. Boenning, M.D., of Philadelphia, 420.

ABSTRACTS AND GLEANINGS.

Influence of Calomel on Fermentation and the Life of Micro-Organisms, 422. Benzoate of Cinchonidine; Treatment of Nævus, 424. Doctor Kocher; Aspidospermo Quebracho; To Hasten the Action of Quinine, 425. A Remarkable Case of Obstetrics,—Abortion at Two Months and Quadruplets at Full Time; Hydrastis in Gonorrhœa; Napthalin in Frostbites, 426. Cancer Remedy; Dr. Martinet; Dr. J. M. DaCosta; Antagonism between Syphilis and Vaccine, 427. Chloral in Albuminuria; Acute Goitre; Amenorrhœa; An Adjuvant to Chloroform, 428. Organic Matter in Drinking Water; The Ownership of Prescriptions; Paraplegia in a Child, 429. Croton-Chloral in the Treatment of Whooping Cough; Iodo-Ferrated Syrup of Coffee, 430.

SCIENTIFIC ITEMS.

Gelatine Test for Organisms in Water; Discovery of Prehistoric Men; Liquid Oxygen and Nitrogen, 431. How to Mix Paints; Strength of Insects, 432.

PRACTICAL NOTES AND FORMULÆ.

Treatment of Catarrh; Iron-rust Spot Remover; Anti-Spree Mixture; Oxide of Zinc in Diarrhœa, 433. Follicular Pharyngitis; Resorcine in the Treatment of Purulent Vaginitis, 434. Eczema Rubrum of the Face and Scalp; Bryonia; On the Treatment of Whooping Cough, 435.

EDITORIALS AND MISCELLANEOUS.

Notice; Death of Dr. Sims; Renewals, 436. Our Next Issue; Will you Help Us? Integrity in Physicians, 437. Transactions, 438. Book Notices; Pampnlets Received, 439. Special Notices, 440.

